# Experiences and Needs of Patients, Caregivers and Nurses during the COVID-19 Pandemic: Study Protocol for a Mixed-Methods Multicentre Study

**DOI:** 10.3390/ijerph191912838

**Published:** 2022-10-07

**Authors:** Colette Balice-Bourgois, Loris Bonetti, Angela Tolotti, Sarah Jayne Liptrott, Michele Villa, Corina Elena Luca, Laura Maria Steiner, Annette Biegger, Silvia Goncalves, Laura Moser, Antonio Palermo, Davide Sari, Dario Valcarenghi

**Affiliations:** 1 Institute of Paediatrics of Southern Switzerland, Ente Ospedaliero Cantonale (EOC), Via Gallino, 12, 6500 Bellinzona, Switzerland; 2 Nursing Research Competence Centre, Ente Ospedaliero Cantonale (EOC), Viale Officina, 3, 6500 Bellinzona, Switzerland; 3 Department of Business Economics, Health and Social Care, University of Applied Sciences and Arts of Southern Switzerland, Via Violino, 11, 6928 Manno, Switzerland; 4 Nursing Development and Research Unit, Oncology Institute of Southern Switzerland, Ente Ospedaliero Cantonale (EOC), Via Gallino, 12, 6500 Bellinzona, Switzerland; 5 Ospedale Regionale di Bellinzona e Valli, Ente Ospedaliero Cantonale (EOC), Via Gallino, 12, 6500 Bellinzona, Switzerland; 6 Cardiocentro Ticino Institute, Ente Ospedaliero Cantonale (EOC), Via Tesserete, 48, 6900 Lugano, Switzerland; 7 Ospedale Regionale di Lugano, Ente Ospedaliero Cantonale (EOC), Via Tesserete, 46, 6903 Lugano, Switzerland; 8 Nursing Department Direction, Ente Ospedaliero Cantonale (EOC), Viale Officina, 3, 6500 Bellinzona, Switzerland; 9 Ospedale Regionale di Locarno, Ente Ospedaliero Cantonale (EOC), Via all’Ospedale, 1, 6600 Locarno, Switzerland; 10 Oncology Institute of Southern Switzerland, Ente Ospedaliero Cantonale (EOC), Via Gallino, 12, 6500 Bellinzona, Switzerland

**Keywords:** COVID-19, pandemic, nursing, patient needs

## Abstract

The COVID-19 pandemic is a major public health problem with millions of confirmed cases and deaths described. Nurses are among the health care professionals most involved at the front line, caring for those affected by COVID-19. Patients and families have been subjected to a high emotional burden of fear, anxiety, and uncertainty. The COVID-19 pandemic has had a significant impact on the approach to patients, the organisation of care, and communication with patients and their families, all requiring considerable adaptation on the part of nurses and health care professionals. The overall aim of this research was to find out the needs of patients with COVID-19, the nursing interventions provided and their outcomes, and to explore the experiences of the nurses, patients, and caregivers. A mixed method study will be performed with a convergent design. The study was divided into three phases. Quantitative methods involved nurses and patients affected by COVID-19 with a questionnaire. Qualitative methods involved nurses, patients, and caregivers with interviews and finally a quantitative analysis of the nursing documentation of the interviewed patients. We hope that this study will help us to understand and identify the main nursing and support needs expressed by patients and their families at different stages of their illness.

## 1. Introduction

The SARS-CoV-2 pandemic represents a major public health problem. At the time of writing, over 455 million confirmed cases and over six million deaths have been reported globally [1].

Nurses are among the health professions most involved at the front line, caring for those affected by COVID-19. Nurses have been subjected to significant demands for sacrifice, in terms of personal risk, increased workload, and difficulty in maintaining the usual standards of care and assistance [2,3]. Health care workers are at high risk of short- and long-term mental health problems, which may impact on the quality of patient care [2,4].

Nursing, centred on personalised and holistic patient care (International Council of Nursing—ICN, 2010), has found itself having to provide exceptional care, finding innovative strategies to ensure the best possible quality, despite sometimes extremely unfavourable conditions. The COVID-19 pandemic has had a significant impact on the approach to patients, the organisation of care, and communication with patients and their families, all requiring considerable adaptation on the part of nurses and health professionals [2,5,6,7].

Patients directly affected by COVID-19 were subjected to notable emotional burdens related to fear, uncertainty, and anxiety, with health care staff having to provide significant support in order to address the patients’ emotions, needs, and experiences. The risks of depression, insomnia, post-traumatic stress disorder (PTSD) and psychological distress are significant for patients and have been described in other infectious disease outbreaks including interventions to address organisational issues [4,8,9,10,11,12,13].

The physical presence of families for hospitalised patients had to be interrupted in order to maintain public safety. Hospitals and health services had to quickly adapt procedures in order to facilitate regular communication between patients and families despite the restrictions. Solutions based on information technologies were introduced and were essential for family-centred care [14,15].

Following previous epidemics (SARS, MERS, H1N1, EBOLA), numerous studies have investigated the experiences of health care professionals, focusing on stress factors present both during and after the pandemic [4,16,17,18,19,20].

These studies were mainly aimed at exploring the experiences of nurses and physicians, while there is limited literature considering the experiences of patients and their relatives. Furthermore, the literature shows that it is always essential to investigate, understand, and consider the needs of patients and their families. This knowledge can help to develop specific nursing interventions to better meet the needs of patients and their relatives [21,22,23,24,25].

Even less research has been conducted to investigate what nursing interventions have been implemented and whether, and to what extent, they have responded to the needs expressed by patients with COVID-19 and their families.

Our study therefore aims to contribute to filling this gap in the literature. The knowledge of these variables will be useful in the case of new pandemics, in order to understand what kind of nursing interventions could be performed, which aspects are effective or ineffective and why, identify areas of best practice and allow for the adoption of improvement strategies where necessary, in order to face similar pandemic situations with greater preparation.

Given the important role nurses play in health emergencies, it would be important to know if and what nursing interventions have been instrumental in ensuring appropriate care and improving patient outcomes.

We would like to clarify that, at the time of this publication, the various research projects contributing to this mixed methods study have been initiated following ethical and regulatory approvals. While data collection has been completed, the strategy for data analysis is outlined within this protocol but has not been completed. For this reason, some sections of the paper are in the past tense, and some are in the future tense.

## 2. Materials and Methods

### 2.1. Research Question, Aim, and Objectives

The overall aim and research question of this study was to find out about the needs of patients with COVID-19, the nursing interventions provided and their outcomes, and to explore the experiences of nurses, patients, and caregivers.

Our objectives were:

(1) To identify the needs expressed by COVID-19 patients and their caregivers/families, whether they were addressed or not and for what reasons, from the point of view of the nurses, patients, and caregivers;
(2) To determine which nursing interventions were performed to address the patients’ needs;
(3) To investigate which interventions were perceived by the patients, caregivers, and nurses to be effective or ineffective;
(4) To determine which factors and/or conditions favoured or hindered the effectiveness of nursing interventions;
(5) To explore the experience of patients and their families during the period of hospitalisation and that of the nurses who cared for them.

### 2.2. Study Setting

This study was performed in a multisite hospital in southern Switzerland. In our country, over three million cases of COVID-19 and 12,000 COVID related deaths have been reported [26].

### 2.3. Study Design

We proposed a mixed methods study, with a convergent design that involved quantitative data collection and qualitative data collection [27]. This design was chosen because the qualitative data allow for further exploration of the results of the quantitative data. The quantitative and qualitative data will be analysed separately and then compared for a more comprehensive understanding of the phenomenon.

The study is divided into three phases (Table 1):

Phase 1: Quantitative investigation of the needs of patients affected by COVID-19, the nursing interventions employed, and their effectiveness through questionnaires to the patients and nurses.

Phase 2: Qualitative investigation of the needs of patients affected by COVID-19, the nursing interventions employed and their effectiveness—data collection from nurses, patients and caregivers.

Phase 3: Quantitative analysis of nursing documentation (electronic patient records). We consulted the electronic patient records of the patients who agreed to be interviewed.

### 2.4. Inclusion Criteria

#### 2.4.1. Nurses

- Nurses who worked in inpatient units where patients affected by the COVID-19 were cared for.

#### 2.4.2. Patients and Family Members

- Adults (aged 18 years or older) who contracted COVID-19 and had a medium to high level of severity of the disease, defined as having been transferred to intensive or semi-intensive care units and after admission to an inpatient unit caring for patients affected by COVID-19. The rationale for this criterion is that it is assumed that such patients have gone through all stages of the disease and have a more complete experience of its different phases.
- The family members of patients (aged 18 years or older) who were willing to be interviewed and who do not have cognitive, psychiatric, or speech problems.
- The participants had to be able to answer a questionnaire and take part in an interview in Italian.

### 2.5. Exclusion Criteria

- Patients diagnosed with cognitive disorders and people with insufficient knowledge of Italian language;
- Individuals who were unable to provide written informed consent to participate in the study.

### 2.6. Data Collection

#### 2.6.1. Phase 1: Quantitative Data

- Nurses

The aim of this phase of research is to determine, from the nurses’ perspective, the main care needs of patients affected by COVID-19 (and their families/caregivers), and the nursing interventions that have been implemented and considered as the most useful in addressing these needs.

The instrument used for data collection was a study specific questionnaire constructed by the research team, and based on Marisa Cantarelli’s Nursing Performance Model (2003) (Table 2) [28,29]. The model considers the person as the bearer of 11 basic needs that, if not satisfied, as in the case of an illness, may require the professional intervention of the nurse through various actions aimed at responding to that specific need.

The usefulness of this model for this investigation lies in the fact that it establishes precise correlations between the needs, professional actions, and expected results. The model is used as a data collection instrument and aggregation system. The additional needs that the questionnaire will assess include the need to maintain cardiovascular function, the need to maintain a safe environment, the need for therapeutic procedures, and the need for diagnostic procedures.

The questionnaire was created and tested by involving nurses who had worked in inpatient units where patients affected by COVID-19 were cared for, in order to obtain feedback on its ability to measure the survey phenomenon. Before being used in the study, the questionnaire underwent pilot testing with five nurses, other than those who were involved in the development phase, in order to assess its clarity and content validity.

The questionnaire also collected demographic variables such as gender, age, years of experience as a nurse, and clinical specialisation. Questions regarding their work experience during the COVID-19 are also included (i.e., if they redeployed to a different inpatient unit from their usual one, if they worked in an ICU ward, when their experience in the COVID-19 ward started, and how long it lasted).

The questionnaire was sent electronically by email to all nurses who worked in COVID-19 wards at the multi-site hospital. At the end of the questionnaire, the respondent was asked if they were willing to participate in an interview.

- Patients

The aim of this phase of research is to find out what needs were expressed by the patients. This should give an overview on which of the patients’ needs were best met and through which professional actions.

The instrument for data collection will include a section of a validated questionnaire, the Newcastle Satisfaction with nursing scale [30], integrated with a study specific section based on nine of the 11 needs of the Professional Performance Model of Marisa Cantarelli (2003) (Table 2). Patient evaluation of the need for diagnostic and therapeutic procedures was excluded for the purpose of this research.

The questionnaire has been tested by five patients to check its clarity and comprehension.

This questionnaire was sent by post to patients who had been treated for COVID-19 who had been admitted to one of the multi-site hospitals and received intensive or semi-intensive treatment and who had showed a prior willingness to participate in research. These were patients who have gone through all phases of the disease including the acute phases. At the end of the questionnaire, the patient was asked if they were willing to participate in an interview as well as a member of their family.

- Recruitment of patients and caregivers

Eligible patients were identified through hospital databases of patients with COVID-19 admitted to COVID wards at the multi-site hospital during the pandemic period, aggregating them by ward. A letter of introduction was sent to the patient, along with an informed consent form (one for the patient and one for the caregiver), the questionnaire to be completed by the patient, and a stamped addressed envelope. Patients were invited to return the completed questionnaire in the envelope provided and state if they were available to be interviewed. Caregivers were also invited to state if they were available to be interviewed.

This was followed by the selection of patients and family members available for the interview and organisation of the interview.

A comparison with the data from the patient investigation may reveal areas of agreement or discordance between the needs of the patients and the interventions implemented by nurses and their perceived effectiveness.

#### 2.6.2. Phase 2: Qualitative Data

- Nurses:

Amongst the nurses who provided their availability, 15–20 subjects were identified for an in-depth interview on the topics of the research. The interviews were semi-structured and digitally recorded. The selection of the professionals was made using the criterion of maximum possible variability [31]. The exact number of interviews with nurses was defined according to the principle of data saturation.

Semi-structured interviews with initial open-ended questions and then with questions more focused on the research question were performed. The thematic areas (professional response to patients’ needs) were the same as the questionnaire, but with the possibility of further investigation and/or clarification to gain an in-depth understanding of the professional response to the patients’ needs. The interview also explored which factors and/or conditions favoured or hindered the effectiveness of nursing interventions. Interviews were performed in a place agreed with the persons concerned (in the hospital or other hospital premises).

- Patients and caregivers:

Among the patients who provided their availability, 15–20 subjects were identified for an in-depth interview on these topics. The interviews were semi-structured and digitally recorded. The selection of patients was made using the criterion of maximum possible variability [31] and who also had family members willing to be interviewed. In this way, it is possible to gain a broad and in-depth understanding of the phenomenon. The number of interviews with family members was defined according to the principle of data saturation.

The instrument used was a semi-structured interview with initial open questions and with later questions more focused on the objectives of the research. The thematic areas (patients’ care needs) were the same as in the questionnaire, but with the possibility of further investigation and/or clarification to gain an in-depth understanding of the professional response to the patients’ needs. The interview with family members was free form and narrative.

The interviews were carried out in a place agreed with the patient and their family (home or hospital) and audio-recorded.

#### 2.6.3. Phase 3: Analysis of Nursing Documentation

The nursing documentation of those patients willing to be interviewed will be analysed. It will be used as an additional source of data to supplement and compare with the results of other sources (interviews and questionnaires).

### 2.7. Data Analysis

#### 2.7.1. Quantitative Data

A descriptive analysis of the participants’ data will be carried out for the main characteristics investigated.

The patients’ needs and nursing interventions will be considered as dependent variables. The characteristics of the patients will be considered as independent variables.

Nominal or categorical variables will be described in terms of the frequency and percentage, ordinal and rational variables in terms of the mean and standard deviation, if normally distributed, or the median and interquartile range, if not normally distributed. The Kolmogorov–Smirnov test will be performed to test the normality of the distributions. If the distributions are not normally distributed, a two-step transformation will be attempted using SPSS 26.0 software, if necessary.

If possible, a multivariate analysis will be performed in order to understand which characteristics of the patients might have influenced their needs.

To compare the nominal or categorical variables, the chi-square or Fisher’s exact test will be used as appropriate.

To compare the ordinal or continuous variables between two groups, the Student’s *t*-test will be used if the variables are normally distributed or a Mann–Whitney test if the variables are not normally distributed.

The analyses will be carried out using SPSS 26.0 software. Statistical significance will be considered with values of *p* < 0.05 [32]

#### 2.7.2. Qualitative Data

The interviews of nurses, patients, and caregivers will be audio recorded and transcribed verbatim. The interviews will be independently analysed by three experienced qualitative researchers and compared. A thematic analysis according to Braun and Clarke will be applied [33]. The analysis of the interviews will be conducted using NVivo 10 software.

The analysis of the nursing documentation will take place after the qualitative phase (as the medical records of the patients involved in the qualitative phase were analysed). The extrapolation of the data from the medical records will be conducted by three people in the research group following the sections of the questionnaire (needs of the persons according to Cantarelli’s model).

To guarantee rigour in qualitative data analysis, the criteria of credibility, transferability, dependability, confirmability by Guba and Lincoln [34], and fittingness by Carnevale [35] will be followed.

#### 2.7.3. Quantitative and Qualitative Data Integration and Rigor in Relational to Mixed Methods Research

We will use joint displays as described in the literature [36,37,38,39] to integrate qualitative and quantitative data. Joint displays are a way to “integrate the data by bringing the data together through a visual means to draw out new insights beyond the information gained from the separate quantitative and qualitative results” [36].

To guarantee rigour, based on the mixed methods literature, we will use the legitimation criteria described by Younas et al., 2020b; Onwuegbuzie A.J. and Johnson R.B. [40,41].

## 3. Conclusions

With this research, we expect to understand the main nursing care and assistance needs expressed by COVID-19 patients at different stages of their illness to uncover which of these needs received adequate professional responses from nurses, and which of the professional actions or interventions were considered as the most useful or effective by those concerned. In the same way, it should be possible to identify some of the needs of patients or their families who, for various reasons, may not have had adequate responses. We believe that this information is important and should be brought to the attention of all professionals in order to be better prepared to deal with similar situations. We hope that this study will help us to understand and identify the main nursing and support needs expressed by the patients and their families at different stages of their illness. The knowledge of the patients’ needs and nursing interventions that were considered useful can be important information to improve patient care in other similar crises, but also during normal care activities.

## Figures and Tables

**Table 1:** The study phases.

	Phase 1	Phase 2	Phase 3
	Quantitative data collection	Qualitative data collection	Quantitative data collection
**Nurses**	Questionnaire	Interview	-
**Patients**	Questionnaire	Interview	Nursing documentation
**Caregivers**	-	Interview	-

**Table 2:** The fundamental nursing needs based on Marisa Cantarelli’s Nursing Performance Model.

Marisa Cantarelli’s Nursing Performance Model
Fundamental nursing needs
Breathing
2. Nutrition and hydration
3. Urinary and bowel elimination
4. Hygiene
5. Movement
6. Resting and sleeping
7. Maintaining cardiovascular function
8. Safe environment
9. Interacting and communication
10. Need for the therapeutic procedures *
11. Need for diagnostic procedures *

* Not included in the patients’ evaluation.

## Data Availability

Not applicable.
